# You Can’t Have AI Both Ways: Balancing Health Data Privacy and Access Fairly

**DOI:** 10.3389/fgene.2022.929453

**Published:** 2022-06-13

**Authors:** Marieke Bak, Vince Istvan Madai, Marie-Christine Fritzsche, Michaela Th. Mayrhofer, Stuart McLennan

**Affiliations:** ^1^ Department of Ethics, Law and Humanities, Amsterdam UMC, University of Amsterdam, Amsterdam, Netherlands; ^2^ QUEST Center for Responsible Research, Berlin Institute of Health (BIH), Charité Universitätsmedizin Berlin, Berlin, Germany; ^3^ School of Computing and Digital Technology, Faculty of Computing, Engineering and the Built Environment, Birmingham City University, Birmingham, United Kingdom; ^4^ Institute of History and Ethics in Medicine, TUM School of Medicine, Technical University of Munich, Munich, Germany; ^5^ ELSI Services and Research, Biobanking and BioMolecular Resources Research Infrastructure European Research Infrastructure Consortium (BBMRI-ERIC), Graz, Austria

**Keywords:** digital health, data access, data privacy, ethics, artificial intelligence, fairness, resource allocation

## Abstract

Artificial intelligence (AI) in healthcare promises to make healthcare safer, more accurate, and more cost-effective. Public and private actors have been investing significant amounts of resources into the field. However, to benefit from data-intensive medicine, particularly from AI technologies, one must first and foremost have access to data. It has been previously argued that the conventionally used “consent or anonymize approach” undermines data-intensive medicine, and worse, may ultimately harm patients. Yet, this is still a dominant approach in European countries and framed as an either-or choice. In this paper, we contrast the different data governance approaches in the EU and their advantages and disadvantages in the context of healthcare AI. We detail the ethical trade-offs inherent to data-intensive medicine, particularly the balancing of data privacy and data access, and the subsequent prioritization between AI and other effective health interventions. If countries wish to allocate resources to AI, they also need to make corresponding efforts to improve (secure) data access. We conclude that it is unethical to invest significant amounts of public funds into AI development whilst at the same time limiting data access through strict privacy measures, as this constitutes a waste of public resources. The “AI revolution” in healthcare can only realise its full potential if a fair, inclusive engagement process spells out the values underlying (trans) national data governance policies and their impact on AI development, and priorities are set accordingly.

## Introduction

The growth of digital health data and increasing computational capabilities have created significant opportunities for the use of artificial intelligence (AI) technology in healthcare. With the ability to learn from large volumes of clinical, -omics, and other health data, AI has the potential to support a wide range of activities: diagnosis, clinical decision making, personalized medicine, clinical research, drug development, administrative processes, and the mitigation of health disparities (Shibata & Wada, 2011; Fleming, 2018; Shortliffe & Sepúlveda, 2018; Davenport & Kalakota, 2019; Fiske, Henningsen, & Buyx, 2019; Liu et al., 2019; Schork, 2019; Woo, 2019). If data-intensive medicine can realize continuous improvement of healthcare quality and thereby reduce patient harm, improve health, empower personal decision making, and increase equity, it would fulfil the core ethical principles of healthcare (ABIM Foundation, 2002; Beauchamp & Childress, 2013; McLennan et al., 2018).

The potential opportunities of AI have led many countries, particularly in the European Union (EU), to invest significant financial and human resources in AI initiatives. In the past few years, previously unseen amounts of public and private investment have flowed into AI applications (KPMG, 2018; CB Insights, 2019). National AI strategies with large, dedicated budgets were published by many EU countries (Righi et al., 2022), e.g., the German federal government promised to allocate 3 billion EUR in funding between 2020–2025 (Die Bundesregierung, 2018). Funding for healthcare and medical AI-related research projects through the EU Horizon 2020 scheme increased between 2014–2020, although large differences in investments can be seen between Member States (around 80 million EUR was awarded to projects in each of the top-funded countries and around 100.000 EUR in countries receiving the lowest amount of funding) (De Nigris et al., 2020, p. 27). To guide the responsible design of these new AI systems in healthcare and beyond, several ethical and legal instruments were newly created by the European Commission (EC), such as the proposed Artificial Intelligence Act (EC, 2021), the Guidelines for Trustworthy AI (EC, 2019), and the updated Medical Device Regulation (EC, 2020), to complement the General Data Protection Regulation (GDPR) which remains the key legal instrument regarding data usage for AI development (EC, 2016).

The use of health data for AI development raises important data privacy concerns, both at an individual and group level (McLennan et al., 2018; Mittelstadt 2019). Thus, there is a tension between incentives and actions that promote AI and incentives and actions that limit access to the required data: “the data hunger of AI runs up against the norm of personal data minimization” (Sorell et al., 2022). This leads to complex dilemmas. All the resources and efforts currently devoted to AI development could go to waste if the issue of data access is not adequately addressed. In this context, it is noteworthy that the proposed EU AI Act requires, for example, the highest levels of data quality and quantity for sufficient training, validation, and testing as well as the necessary heterogeneity to cover relevant patient (sub)populations and variants in the intended clinical setting (Art. 10). This requires broad access to healthcare data, and tools not fulfilling these requirements would not be permitted. Countries must thus decide how to balance the positive goals of secondary-use activities like healthcare AI with mitigating associated privacy risks. These trade-offs raise issues of resource allocation and justice that have so far been largely neglected in policy debates and the scholarly literature. In this perspective article, we provide an overview of these macro-level ethical trade-offs related to data use for healthcare AI. While we remain neutral on how one should value data privacy and access, we conclude by providing procedural recommendations that allow this decision to be made in a fair manner.

## Variation in European Union Data Governance

Health-related AI applications are in crucial need of patient data during the development of the AI model in the training, validation and test phases. These health data are often initially collected for a different purpose than AI development, and this secondary use requires a valid ethical and legal basis. In Europe, the central legal instrument in this domain remains the GDPR which is directly enforceable in all EU Member States and applies to all EU citizens. The GDPR has the dual aim of protecting personal data, meaning data that can be traced back to living individuals without unreasonable effort, and achieving a higher level of harmonization of data protection practices.

As a result of political compromises, however, the GDPR leaves it open in several places for Member States to issue derogations in their national law when it concerns public interest, scientific or historical research purposes or statistical purposes. (Heckmann and Scheurer, 2021). This may include deciding on what constitutes sufficient methods of pseudonymization, when data can be considered fully non-identifiable, what further restrictions should be imposed on processing sensitive data for research purposes, and what are sufficient safeguards and conditions for processing data under the research exemption (Shabani et al., 2018). In addition to the GDPR, national health and biobanking laws might also have implications for data protection requirements and ultimately access to health data and data governance. (Bak et al., 2020; Kindt et al., 2021; Slokenberga et al., 2021) As a result, there remains a wide variation of data governance approaches across Europe and the actual balance between data protection rules and access requirements is struck at country-level. In this regard more conservative Germany and more liberal Finland are examples of countries that differ in their approaches to data governance.

The Finnish approach to data access is evident in its Act on the Secondary Use of Health and Social Data (Ministry of Social Affairs and Health, 2019) which provides the basis for the national data permit authority FinData to facilitate access to and sharing of patient data. The country has adopted a national policy oriented towards big data and open data to transform the technical and governance infrastructure for AI and other computer science research (Aula, 2019). In Finland, consent is not legally required for including personal data in national health registries, but data access is controlled through detailed policies and security procedures (Vrijenhoek et al., 2021). Moreover, the Biobank Act (2012) which is currently undergoing further reform, allows samples and related data to be used for research purposes without (re-)consent for every research project, and biobank samples can be linked to health data from national registries. Being the frontrunner in developing a national AI strategy already in 2017, Finland is among the most digitally developed EU countries and provides an online service which citizens use to view their health information from different sources (EC, 2019; Jormanainen et al., 2019). There is an explicit focus on public education and awareness, including a free online AI course. As in other Nordic countries, Finland´s national AI strategy generally reflects the core values of trust, openness, and transparency (Robinson, 2020).

This contrasts with the German approach that has traditionally been geared toward comprehensive control and where health data research is usually conducted with patient consent. For example, consent is *the* legal basis for any processing of data stored in the newly launched electronic patient record (elektronische Patientenakte or ePA) whose use is voluntary, and which gives patients full control over their data (Molnar-Gabor et al., Forthcoming 2021). Data processing for scientific research in the public interest might take place without consent, if organizational and technical provisions are met, as specified in the Federal Data Protection Act (Molnár-Gábor et al., 2018). In 2018, the German State Minister for Digitalization stated that the country’s strict data protection laws block development in the healthcare sector (Kaiser, 23 December 2018). Indeed, in practice, this research exemption seems hardly ever used. A recent interview study with researchers, data protection officers and research ethics committee representatives in the state of Bavaria, found that German law was perceived as vague and was differently interpreted across federal states and institutions (McLennan et al., 2022a). This resulted in secondary health data research usually only taking place when consent had been obtained or data were fully anonymized.

## Trade-Offs in Realizing the Potential of Artificial Intelligence in Healthcare

### Data Privacy Versus Data Access

This variation in data governance approaches can hamper (inter-)national data sharing and makes it difficult to create disease registries and to develop AI tools (De Lange et al., 2019; McLennan et al., 2019; Haneef et al., 2020). The disagreement over the interpretation of certain provisions in the GDPR, including research exemptions, is not easily solved as it stems from different viewpoints on how to balance foundational values like informational self-determination versus solidarity (Hoffman et al., 2012; van Veen, 2018). Whether (national) strategies should focus on data privacy or data access is a difficult question linked to various ethical dilemmas. Namely, what we might identify as a more liberal approach to data access might have in turn serious implications for fundamental rights to privacy. A restrictive approach, on the other hand, might undermine data-intensive medicine and in turn cause harm by biasing models and leading to wasted investments into AI development.

Governments and institutions taking a more liberal approach to data governance, i.e., interpreting the GDPR generously by focusing on its harmonization and data sharing aim, may face complex ethical issues and public resistance. For instance, the *care.data* program in the United Kingdom famously collected health data for secondary use without informed consent and with limited options for opt-out, which adversely affected public trust in health data initiatives (Vezyridis & Timmons, 2017). Innovations in AI may promise to improve the quality of care and lower costs, but the need for detailed personal information as input data exacerbates known concerns about issues like data privacy, bias and discrimination (Mittelstadt & Floridi, 2016; Price & Cohen, 2019).

Those with a more restrictive view on data governance generally use the “consent or anonymize” mind set: personal data may only be used if informed consent is obtained or the information is fully anonymized (Mostert et al., 2016). However, requiring (re-)consent can lead to significant administrative and financial hurdles that delay important activities or even make them unfeasible (Tu et al., 2004; Jansen et al., 2007). Requiring (re-)consent may also lead to major selection biases that undermine data representativeness, which can lead to biased AI models that in turn harm patients and exacerbate existing health inequalities (Vayena et al., 2018). In addition, while consent may protect the privacy of persons whose data are used to train and test AI models (that is, if the information is clear and unambiguously presented and the patient is in a position to make a reasoned decision), it does not protect the privacy of others who did not consent but can still have inferences drawn about them based on rules derived from a cohort of consenting individuals (Barocas et al., 2014).

Furthermore, although anonymized data is out of scope of the GDPR, data anonymization is not free of technical, legal and ethical challenges. Full anonymization has become increasingly difficult due to the potential of cross-linking datasets and the inclusion of highly personal data like genetic sequences (Gymrek et al., 2013). Further, irreversible anonymization may involve removing essential information needed to perform secondary activities like research. Additionally, some authors argue that anonymization is merely possible in a specific context for a short period of time and requires regular reassessments to determine whether the status of anonymization can still be upheld, making it equally resource intense as asking consent (Sariyar and Schlünder, 2016). Even if full anonymization was possible and/or feasible, it offers no guarantees that AI models based on such “anonymous” data do not harm the individuals who donated their data (Barocas et al., 2014).

In Europe, concerns have been raised for several years about the “overprotection” of personal data under (draft versions of) the GDPR, which are still relevant given the varying interpretations of the regulation (Ploem et al., 2013; Author Anonymous, 2015; Timmers et al., 2019). In a recent open letter by genetic researchers, a similar concern was voiced about access to digital sequence information that can be used for public health, as policy negotiations are feared to favour data sovereignty and limit data sharing under the Convention on Biological Diversity (DSI, 2022). The broader debate on informational self-determination versus scientific data research dates back well into the previous century. Yet, when it comes to AI, we sometimes seem to forget that data access is the most important prerequisite for any AI innovation. This omission may lead to a situation where some policies follow the current trend of pouring tremendous resources into health AI developments when, at the same time, the success of the funded research is effectively made impossible due to the country´s specific interpretation of the GDPR and relevant national law.

### Overprotection or Overinvestment?

The potential of healthcare AI in Europe is limited when countries’ data governance approaches are overly strict, ambiguous, or contradicting. Haneef et al. (2020) surveyed the use of AI by national public health institutes and found it limited in practice, reportedly due to the complexity of data regulation laws coupled with lack of human resources and the absence of a robust data governance framework in various countries and institutions. Enabling researchers to create AI applications that help improve care, requires giving them greater access to patient data, albeit conditional and within a secure environment. The EU and several Member States seek to achieve a win on all fronts, i.e., they want to become both a leading player in health AI as well as provide maximum protection regarding health data privacy. However, policy-makers must realize that hard choices are unavoidable to be able to strike the right balance in data governance.

Public resources are generally finite, so whether the right to health is best fulfilled by prioritizing investment in AI-driven technologies over data infrastructure development or other healthcare spending, is ethically relevant. As we indicated above, a country that takes a very restrictive approach to data access needs to take this into account when allocating funds. Future legislation such as the proposed EU AI act could essentially ban AI in healthcare applications if developers do not have broad access to relevant healthcare data and therefore cannot meet generalization and bias mitigation requirements. Thus, development of robust technological data management and governance structures, such as the proposed European Health Data Space (EHDS) and standards for interoperability of health records that promise to improve data access and usability (Shabani, 2022), should then be established prior or at least in parallel to the creation of specific AI tools. The European Investment Bank claims that the EU is limiting innovation by underinvesting in AI, quoting an investment gap of up to 10 billion EUR (Verbeek & Lundqvist, 2021), but we disagree with this general statement. Rather, investing in AI-driven healthcare technology that cannot prosper due to unresolved data governance issues would rather constitute an overinvestment, i.e., an unjust waste of resources.

Moreover, resources allocated to health AI may come at the expense of non-AI solutions. Since the value of AI remains uncertain and many health interventions in the field of AI are—thus far—of limited real-world effectiveness (D’Amour et al., 2020; Skorburg et al., 2021), it has been argued that policy-makers should not allocate resources to AI tools exclusively, especially when these resources could strengthen existing evidence-based solutions and help to overcome structural barriers to care (Skorburg et al., 2021). This dilemma is well-known in the field of public health. For instance, in the field of HIV prevention in low- and middle-income countries, the development of pharmaceutical PrEP (Pre-Exposure Prophylaxis) led to fears that funding for the free provision of condoms would be curtailed. However, PrEP, was never intended to be a stand-alone intervention and its combined use with condoms has proven to be more effective and acceptable than either intervention on its own (Bak et al., 2018). Similarly, the discussion around AI in medicine has shifted away from the complete replacement of physicians and their judgement to more synergistic uses of AI (i.e. doctors plus AI) (Mazzanti et al., 2018; Dos Santos et al., 2019). Thus, if actors decide to invest in health AI, this needs to be accompanied with investment into not only data access structures but also the surrounding healthcare system that interacts with the AI tool. Nonetheless, this might be difficult given resource constraints. How then should we decide what constitutes just resource allocation for health AI?

## Towards a Fair Prioritization for Health Artificial Intelligence

Most of the literature on AI ethics focuses only on the fairness concerns *inherent* to this upcoming technology (e.g., related to bias and discrimination in the models), rather than on the trade-offs between data privacy and access and the resulting questions of resource allocation. For example, in the high-level expert guidance on Trustworthy AI by the EC, seven key requirements are listed that should be implemented by model developers and about which end-users should be informed (EC, 2019). By emphasizing the requirements of the AI system itself, however, the EC narrows the ethical debate to the interaction with a specific application. While such principlist guidelines can help sensitize professionals to the built-in values of AI applications, they do not provide a solution to the wider moral dilemmas that arise from value conflicts and resource limitations (Bak, 2020).

**FIGURE 1:**
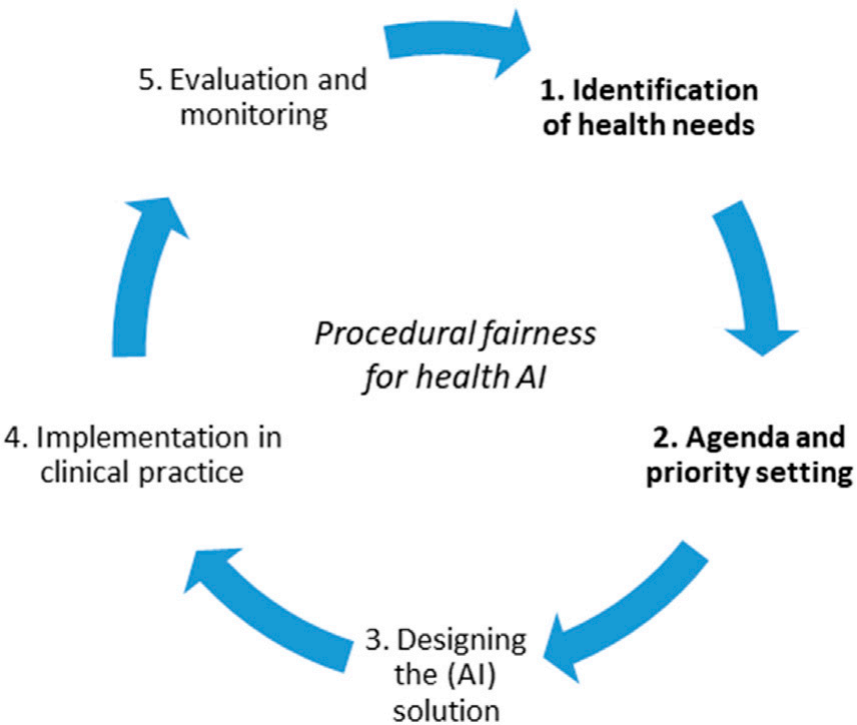
Procedural fairness for priority-setting in health AI, with special attention for steps 1 and 2. Adapted from the Policy Cycle (Howlett & Giest, 2015).

Discussions about ethical requirements for AI should thus be preceded by a broader ethical debate about these priorities: rather than just holding AI to account, our public investments in AI should be accountable. The policy and planning cycle of health intervention development helps illustrate our point (Figure 1). While the focus of most ethicists and policy-makers has been on step 3 (the design of the AI solution) and to a lesser extent steps 4 and 5 (implementation and evaluation), we want to refocus the debate on steps 1 and 2 of the cycle (identification of health needs and subsequent priority-setting). Our suggestion is in line with recommendations from the World Economic Forum that the creation of national AI strategies should start with a SWOT (strengths, weaknesses, opportunities, and threats) analysis, as was done in Finland, to keep policy goals in line with resource constraints and needs of citizens (Madzou et al., 2019). This is ultimately a political discussion, as is any debate on technology that involves choices between competing values.

The conditions of such societal debate can be found in the work of the American philosopher Norman Daniels (2007), who argues that when there is no consensus on substantive values, we should focus on procedural values. Fair process is important as it allows healthcare organizations to pursue their (research) policies with a mandate from society. This idea was formalized into a model known as Accountability for Reasonableness (A4R) which proposes key conditions for the legitimacy of decision-making in public health (Daniels & Sabin, 1997). It is beyond the scope of this paper to discuss the A4R framework in detail but it has been found valuable for the field of digital health (Wong, 2020) and was used for drafting the Montreal Declaration for Responsible Development of AI, which launched in 2017 after an extensive public deliberation process (Dilhac et al., 2018; Brall et al., 2019). We support the idea that A4R or similar procedural fairness frameworks should be used in deliberations about resource allocation for health AI.

Decision-makers in EU countries should structurally engage an inclusive group of researchers, data subjects, clinicians, and other relevant stakeholders, to deliberate the trade-offs between data privacy and the value of AI. We want to emphasize we do not suggest favouring any of the two approaches but propose that inclusive engagement or “data democracy” is needed to ensure that decisions empower affected communities and are sensitive to their specific needs, which in turn may help to promote public trust (Ienca et al., 2018; Kalluri, 2020; Nyrup, 2021). Ethicists may join the process to help explain and clarify complex moral questions (McLennan et al., 2022b). This of course requires transparent insight into the available budgets and competing needs. All in all, if such reflections lead to a country explicitly deciding to focus on a strict, conditional or liberal approach to data privacy and/or data access, that decision is morally legitimate if it fulfils conditions of procedural fairness, e.g. accountability and transparency.

## Conclusion

The development and implementation of AI for healthcare comes with trade-offs: striving for all-embracing data privacy has proven incompatible with the desire to realize the full potential of AI for medical purposes. We have outlined that countries need to implement digital health strategies that are consistent, which requires an examination of the core values that underlie the national data governance frameworks. In a nutshell, they should deliberate with their citizens and be able to explain to them why they have set certain priorities, and the chosen balance between specific data privacy and data access conditions should be reflected in the national and ultimately European AI budgets. Failing to do so is leading to distributive justice concerns that should not be overlooked in debates on the ethical aspects of health-related AI.

## Data Availability

The original contributions presented in the study are included in the article/Supplementary Material, further inquiries can be directed to the corresponding author.
